# Distinct Mitochondrial Remodeling During Mesoderm Differentiation in a Human-Based Stem Cell Model

**DOI:** 10.3389/fcell.2021.744777

**Published:** 2021-10-14

**Authors:** Sepideh Mostafavi, Novin Balafkan, Ina Katrine Nitschke Pettersen, Gonzalo S. Nido, Richard Siller, Charalampos Tzoulis, Gareth J. Sullivan, Laurence A. Bindoff

**Affiliations:** ^1^Department of Clinical Medicine, University of Bergen, Bergen, Norway; ^2^Division of Psychiatry, Haukeland University Hospital, Bergen, Norway; ^3^Norwegian Centre for Mental Disorders Research (NORMENT)—Centre of Excellence, Haukeland University Hospital, Bergen, Norway; ^4^Institute for Biomedicine, University of Bergen, Bergen, Norway; ^5^Neuro-SysMed, Center of Excellence for Clinical Research in Neurological Diseases, Department of Neurology, Haukeland University Hospital, Bergen, Norway; ^6^Stem Cell Epigenetics Laboratory, Institute of Basic Medical Sciences, University of Oslo, Oslo, Norway; ^7^Department of Molecular Medicine, Institute of Basic Medical Sciences, University of Oslo, Oslo, Norway; ^8^Norwegian Center for Stem Cell Research, Oslo University Hospital and the University of Oslo, Oslo, Norway; ^9^Institute of Immunology, Oslo University Hospital, Oslo, Norway; ^10^Hybrid Technology Hub—Centre of Excellence, Institute of Basic Medical Sciences, University of Oslo, Oslo, Norway; ^11^Department of Pediatric Research, Oslo University Hospital, Oslo, Norway

**Keywords:** mitochondria, development, metabolism, stem cells, cardiomyocyte, OXPHOS

## Abstract

Given the considerable interest in using stem cells for modeling and treating disease, it is essential to understand what regulates self-renewal and differentiation. Remodeling of mitochondria and metabolism, with the shift from glycolysis to oxidative phosphorylation (OXPHOS), plays a fundamental role in maintaining pluripotency and stem cell fate. It has been suggested that the metabolic “switch” from glycolysis to OXPHOS is germ layer-specific as glycolysis remains active during early ectoderm commitment but is downregulated during the transition to mesoderm and endoderm lineages. How mitochondria adapt during these metabolic changes and whether mitochondria remodeling is tissue specific remain unclear. Here, we address the question of mitochondrial adaptation by examining the differentiation of human pluripotent stem cells to cardiac progenitors and further to differentiated mesodermal derivatives, including functional cardiomyocytes. In contrast to recent findings in neuronal differentiation, we found that mitochondrial content decreases continuously during mesoderm differentiation, despite increased mitochondrial activity and higher levels of ATP-linked respiration. Thus, our work highlights similarities in mitochondrial remodeling during the transition from pluripotent to multipotent state in ectodermal and mesodermal lineages, while at the same time demonstrating cell-lineage-specific adaptations upon further differentiation. Our results improve the understanding of how mitochondrial remodeling and the metabolism interact during mesoderm differentiation and show that it is erroneous to assume that increased OXPHOS activity during differentiation requires a simultaneous expansion of mitochondrial content.

## Introduction

Crosstalk between mitochondria, metabolism, and processes controlling stem cell fate is essential (reviewed in Wanet et al., 2015). As human pluripotent stem cells (hPSCs) exit pluripotency, they switch from a primarily glycolytic-based metabolism that provides the energy and substrates necessary for proliferation in a hypoxic niche to one more dependent on oxidative phosphorylation (OXPHOS), which is better suited for post-mitotic tissues with high energy demand (Locasale and Cantley, 2011; Varum et al., 2011; Zhang et al., 2012a). Metabolic switching during differentiation is thought to be germ layer specific, as inhibition of glycolysis inhibits neuronal differentiation but has no effect on mesodermal and endodermal differentiation (Cliff et al., 2017). In addition, the MYC transcription factor family that drives glycolysis remains activated after exit from pluripotency in nascent ectoderm, while it is silenced during mesoderm and endoderm differentiation (Cliff et al., 2017). How mitochondrial properties are adapted through metabolic switching during early stages of differentiation across different germ layers remains unclear.

It has been proposed that the switch from glycolysis to OXPHOS is the result of remodeling mitochondria from a fragmented state with lower mitochondrial DNA (mtDNA) and mass in hPSCs, to elongated mitochondria with high levels of mtDNA and mitochondrial mass in differentiated cells (Sercel et al., 2021). Experimental data for this hypothesis are, however, limited to either studies of neuronal differentiation (Zheng et al., 2016; Lees et al., 2018) or to earlier studies describing mitochondrial changes in heterogeneous cultures derived from spontaneous differentiation of embryoid bodies (St John et al., 2005; Cho et al., 2006; Belmonte and Morad, 2008; Lukyanenko et al., 2009). Notably, ectoderm differentiation showed a biphasic change in mitochondrial content: in early differentiation, mitochondrial mass, mtDNA, and superoxide production decreased, while in the later stages, there was an expansion of mitochondrial mass and mtDNA along with a higher OXPHOS activity (St John et al., 2005; Cho et al., 2006; Zheng et al., 2016; Lees et al., 2018).

Understanding the crosstalk between mitochondrial remodeling and metabolic switch is particularly crucial for improving our knowledge about the mechanisms involved in lineage-directed differentiation and tissue maturation (Wanet et al., 2015). To gain greater insight into the role of mitochondrial remodeling during metabolic switch in mesoderm differentiation, we assessed mitochondrial properties including mitochondrial abundance, ultrastructure, membrane potential, and respiratory complex activity during differentiation and maturation of cardiomyocytes derived from both human embryonic stem cells (hESCs) and human induced pluripotent stem cells (hiPSCs). Cardiomyocytes derived from hPSCs share the characteristics and functional properties of primary human heart tissue and are able to recapitulate the *in vivo* developmental process (Zwi et al., 2009; Zhu et al., 2017; Friedman et al., 2018).

In contrast to previous reports, we detected a significant reduction in mitochondrial biomass and mtDNA levels during mesoderm differentiation. Despite this marked mitochondrial reduction, however, differentiated cells showed a higher mitochondrial coupling efficiency and appeared more dependent on OXPHOS with a higher mitochondrial membrane potential per unit mitochondria than undifferentiated hPSCs. Overall, our findings suggest a unique mitochondrial remodeling process for cardiomyocyte differentiation whereby mitochondrial biogenesis decreases during the transition from hPSCs into differentiated cardiomyocytes, while the efficiency of ATP generation through OXPHOS increases in keeping with mitochondrial maturation.

## Materials and Methods

### Human Pluripotent Stem Cell Lines

Three hESC lines [H1 (male), 429 (female) and 360 (male)] and four hiPSC lines (established from two independent Detroit 551 (female) clones (clones 7 and 10) and one CRL 2097 (male) iPSC clone, CRL-8) were employed for this study. The details of reprogramming and characterization of the hiPSC lines are published elsewhere (Siller et al., 2015, 2016; Balafkan et al., 2020; Liang et al., 2020). The hPSCs were cultured using standard procedures in a 5% CO_2_ incubator at 37°C. hPSCs were maintained in feeder-free conditions in Essential 8 Medium (E8) (Gibco™). The passage number of the hiPSCs was between 23 and 55 for all experiments, and there was no preference for using a specific passage number for an experiment.

### Cardiomyocyte Differentiation

Cardiomyocyte differentiation was performed in 96-well microplates as previously described (Balafkan et al., 2020). Briefly, hiPSCs were seeded and propagated on Geltrex (Gibco™), under feeder-free conditions in E8. Within 3 days, when cells reached the optimum confluency (60–70%), cardiomyocyte differentiation was started by applying the GSK3 inhibitor CHIR99021 in Roswell Park Memorial Institute (RPMI) 1640 medium supplemented with B27 without insulin (RPMI-B27), in a cell-concentration-dependent manner. After 24 h, medium was changed to RPMI-B27 without CHIR99021. The differentiation process was continued by adding 5 μM of inhibitor of WNT production-2, IWP2, diluted in RPMI-B27, 72 h post-differentiation induction for 48 h. Fresh RPMI-B27 medium was provided on day 5, and from day 7, cells were fed with fresh RPMI medium supplemented with B27 with insulin without extra supplement, every 2 days. Differentiated cardiomyocytes start beating around day 10, and it becomes more synchronized after day 12 (see Supplementary Movies 1, 2).

### Gene Expression Analysis

MagMAX^TM^-96 Total RNA Isolation Kit was used for RNA isolation from cultured cells. EXPRESS One-Step Superscript qRT-PCR Kit (Invitrogen™) was used for cDNA synthesis and real-time PCR using TaqMan probes (Supplementary Table 1) on an Applied Biosystems 7,500-Fast real-time PCR System. All real-time PCR were performed in triplicate, and the average of Ct values was normalized to the geometric mean of *ACTB* and *GAPDH* as endogenous control genes, and the result (dCt) used for further analysis. For a detailed description, see the Supplementary Material.

### RNA Sequencing and Bioinformatics Analysis

RNA sequencing (RNA-seq) analyses were performed independently for two datasets. The first dataset (Dataset A, *N* = 22) was composed of either hESC or hiPSC lines collected from S1 (undifferentiated cells at day 0) and S4 (Isl1^+^ progenitor cells at day 7), from three independent differentiation runs of two hESC lines (429 and 360), two independent differentiation runs of Detroit-7 and CRL-8, and one of Detroit-10. The second dataset (Dataset B, *N* = 12) was composed of samples collected at four stages spanning S1–S5 (specifically days 0, 2, 5, and 15) from H1. RNA-seq sample information is provided in Supplementary Tables 3, 4. Differential gene expression analyses were performed using the DESeq2 R package version 1.26 (Love et al., 2014) with default parameters, using cell line as a covariate in the model for the first dataset. A corrected *p*-value of 0.05 and log_2_ (fold change) of 1 were set as the threshold for significantly differential gene expression. Genes then were tested for enrichment using the gene score resampling method implemented in the ermineR package version 1.0.1, an R wrapper package for ermineJ (Gillis et al., 2010) with the complete Gene Ontology (GO) database annotation (Ashburner et al., 2000), and the Kyoto Encyclopedia of Genes and Genomes (KEGG) database (Kanehisa and Goto, 2000) to obtain lists of upregulated and downregulated pathways for each cohort. The source code for the RNA-seq analyses is available in the GitLab repository^1^ under GPL public license v3.0. For a detailed description of the methods, refer to the Supplementary Material.

### Flow Cytometry

Cells were dissociated into single-cell suspension using TrypLE^TM^ Express Enzyme (Gibco™) and stained with Zombie Red^TM^ Fixable Viability Kit (BioLegend, San Diego, CA, United States) according to the manufacturer’s instructions. Single cells were fixed, blocked, and stained accordingly (for a detailed description, see the Supplementary Material). Antibodies are listed in Supplementary Table 1. At least 30,000 events were collected for the target marker using a Sony cell sorter SH800 (Sony Biotechnology Inc., San Jose, CA, United States), and collected data were analyzed and presented by FlowJo V.10.5.0 (FlowJo LLC, Ashland, OR, United States). To conduct inter- and intra-sample analyses from different runs and minimize the batch-to-batch variation, the flow cytometer was calibrated prior to quantitative fluorescence intensity measurements using Quantum^TM^ Alexa Fluor^®^ 488 MESF (molecules of equivalent soluble fluorophore; Bangs Laboratories, Fishers, IN, United States), and collected median fluorescence intensity (MFI) was normalized to MESF as an external control. All gates were adjusted according to fluorescence minus one control (FMO).

### Immunocytochemistry and Fluorescence Microscopy

Cells were seeded either on Geltrex-coated cover slips or in Millicell^®^ EZ SLIDES (Merck Millipore, Billerica, MA, United States), fixed, and stained accordingly (for a detailed description, see the Supplementary Material). A final concentration of 10 μg/ml was used for all primary antibodies, and they were incubated overnight at 4°C. Confocal microscopy images were taken on either a Zeiss LSM 510 META (Carl Zeiss, Oberkochen, Germany) or a Leica TCS SP5 (Leica Microsystems GmbH, Wetzlar, Germany) at the Molecular Imaging Center (MIC), University of Bergen; and data analysis and image editing were done with Fiji (Schindelin et al., 2012). Antibodies are listed in Supplementary Table 1.

### Transmission Electron Microscopy

Samples were prepared by the MIC, Department of Biomedicine, University of Bergen; and grids were imaged using a Jeol JEM-1230 transmission electron microscope (TEM) at 80 kV. For a detailed description, see the Supplementary Material.

### Mitochondrial DNA Analysis

MtDNA quantification and deletion assessment were performed on DNA isolated from cultured cells using MagMAX^TM^-96 DNA Multi-Sample Kit (Invitrogen™) and real-time PCR, as well as long-range PCR, as previously described (Tzoulis et al., 2013). A commonly deleted region (MT-ND4) in the major arc of mtDNA and a rarely deleted region (MT-ND1) were utilized to quantify deletion, and MT-ND1 was compared with amplification of a single-copy nuclear gene (APP) to assess the number of mtDNA copies. For a detailed description (see Supplementary Material).

### Measurement of Oxygen Consumption Rate and Extracellular Acidification Rate Using Seahorse XF^e^96 Analyzer

Respiration and acidification rates were measured on a monolayer culture of undifferentiated hPSCs and cells at S5 using a Seahorse XF^e^96 extracellular flux analyzer (Agilent, Santa Clara, CA, United States). For a detailed description (see Supplementary Material). To correct the final results for differences in cell size and number between the undifferentiated hPSCs and differentiated cells at S5, we measured the total protein concentration for each well using absorbance at 280 nm. All results were reported as pmol O_2_ per min after normalization to total protein concentration. The XF reader software (Wave Desktop 2.4) was used to analyze the data.

### Measurement of Mitochondrial Membrane Potential

We used a previously described protocol (Rowe and Boletta, 2013) to analyze the mitochondrial membrane potential (ψm) independent of cell volume using tetramethylrhodamine methyl ester (TMRM). We quantified the MFI of TMRM before and after applying the uncoupler carbonyl cyanide 4-(trifluoromethoxy)phenylhydrazone (FCCP) to dissipate the mitochondrial membrane potential. The difference between geometric means for TMRM- and TMRM–FCCP-treated cells provided the membrane potential normalized to cell size. In addition, to normalization for mitochondrial mass differences, we calculated the ratio of median MFI of TMRM to MFI of TOM20, so that we could express the level of mitochondrial membrane potential per unit of mitochondrial membrane area measured. For a detailed description (see Supplementary Material).

### Statistical Tests

Prior to assessing statistical significance, and in order to identify the appropriate statistical test, we tested the normal distribution of the sample population using the Shapiro–Wilk and Kolmogorov–Smirnov tests. Parametric tests were only used for datasets that passed both tests. Of note, all flow cytometry data were treated as non-normally distributed populations. Supplementary Table 2 demonstrates the statistical tests used for each assay to assess statistical significance. N represents the number of biological replicates. Each hPSC line is one biological replicate, and each round of differentiation from a given hPSC line represents one technical replicate. Data were analyzed and plotted using GraphPad-Prism (Prism 7.0, GraphPad Software, La Jolla, CA, United States).

## Results

### Cardiomyocyte Differentiation and Characterization

**FIGURE 1:**
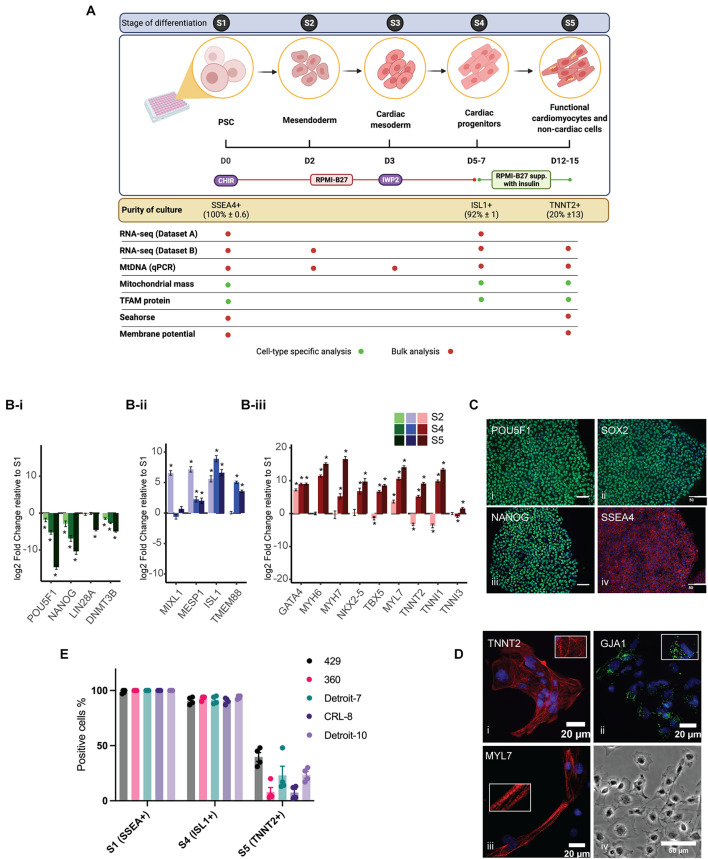
Cardiomyocyte differentiation and characterization. **(A)** A schematic diagram showing the cardiomyocyte differentiation protocol. The differentiation process was divided into five stages (S1–5), shown in the top panel. hiPSCs were treated with 6 μM and hESCs with 8 μM of CHIR99021 (CHIR) on day 1 for 24 h followed by treatment with IWP2, a Wnt signaling inhibitor, for 48 h on day 3 in RPMI supplemented with B27 without insulin. By S4, medium was changed to RPMI supplemented with B27 with insulin. Culture purity was assessed with flow cytometry with stage-specific cell markers (in yellow box). The table shows which experiment was conducted for each stage and whether we assessed a heterogeneous culture (bulk analysis, red dots) or cell-type specific format (green dots). Schematic diagram is created with BioRender.com. **(B)** Relative expression of known markers for stem cells **(B-i)**, cardiac progenitor **(B-ii)**, and cardiomyocytes **(B-iii)** were assessed using RNA-seq data from H1 (Dataset B). **(C)** hPSCs examined for expression of stem cell-specific markers using a panel of antibodies: POU Class 5 Homeobox 1 (POU5F1, also known as OCT4), SRY-box 2 (SOX2), NANOG Homeobox (NANOG), and stage-specific embryonic antigen-4 (SSEA4). Scale bars demonstrate 50 μm. **(D)** hPSC-derived cardiomyocytes were stained for cardiomyocyte-specific markers: cardiac muscle troponin T-type 2 (TNNT2), Gap Junction Protein Alpha 1 (GJA1), and Atrial Light Chain-2 (MYL7). Pictures show co-immunostaining of the cell-specific markers (labeled on the image) and nucleus (blue). **(D-iv)** A bright field image of cardiac cells at S5 (transferred from a 96-well plate to cover glass at S4). **(E)** The bar charts represent the purity of cultures at three critical stages of mesoderm differentiation and the variation among the lines. Each dot represents a single differentiation; error bars are the SD of the mean. hiPSCs, human induced pluripotent stem cells; hESCs, human embryonic stem cells; RPMI, Roswell Park Memorial Institute; hPSCs, human pluripotent stem cells.

Two hESC lines (429 and 360) and three hiPSC lines (established from two independent Detroit 551 clones (clones 7 and 10), and one CRL 2097 iPSC clone, CRL-8) were selected for mesoderm differentiation in a 96-well plate format (Balafkan et al., 2020). The differentiation process is shown schematically in Figure 1A. For comparative purposes, we divided the differentiation process into phases based on the expression of cell-type-specific markers (Figure 1A): pluripotent state (S1, day 0), mesendoderm cells (S2, day 2), cardiac mesoderm (S3, day 3), Isl1^+^ progenitor cells (S4, days 5–7), and cardiomyocyte and non-cardiac cells (S5, days 12–15). The specific markers for the mesoderm cell lineage were assigned based on previous studies (Sturzu and Wu, 2011; Vliet et al., 2012). Initial transcriptomic profiling of Isl1^+^ progenitor cells (dataset A) derived from different hPSCs was performed with samples collected from four hiPSCs and two of the hESC lines (429 and 360) at pluripotent state (S1) and Isl1^+^ progenitor cells (S4). Sample clustering based on the RNA-seq gene expression evidenced a marked transcriptional difference between S1 and S4, singling out the differentiation process as the main driver of transcriptional change (Supplementary Figure 1A). This was corroborated by the strong association between the first principal component of gene expression (explaining 36% of the variance) and the differentiation stage (linear regression model *p* = 71 × 10^–3^). Cell lines were only weakly associated with subsequent principal components (Supplementary Figure 1B). To validate our gene expression analysis and further profile transcriptomes from critical stages of mesoderm differentiation, including S5, an independent RNA-seq experiment was performed using an hESC line (H1), which was solely used for transcriptomic analysis (dataset B) and not included in other sets of experiments. RNA samples from three independent differentiations of H1 were collected at the following stages: pluripotent state (S1), mesendoderm state (S2), cardiac progenitor (S4), and differentiated cells in S5. Both datasets revealed downregulation of pluripotent stem cell markers, while upregulated genes were enriched in pathways associated with cardiomyocyte differentiation including regulation of cardiac muscle, ventricular cardiac muscle tissue morphogenesis, sarcomere, and regulation of heart contraction (Figure 1B and Supplementary Figures 1C–E). The correct mesoderm differentiation route was confirmed using qPCR (Supplementary Figure 2A) and immunocytochemistry (Figures 1C,D). These data indicate that our model is a reliable model in which to assess mesoderm differentiation toward cardiomyocytes. Previously, we have shown that differentiating cells in a 96-well plate format results in a population of both cardiomyocytes and non-cardiomyocytes (Skelton et al., 2017; Balafkan et al., 2020). Thus, cells in S1 represent a pure population of PSCs (∼100% ± 0.6 SSEA4^+^), and those in S4 represent a relatively pure population of cardiac progenitors (∼92% ± 1 ISL1^+^) (Moretti et al., 2006), while S5 comprises two different cell populations, cardiomyocytes (20% ± 13 TNNT2^+^) and non-cardiomyocyte (TNNT2^–^) cell populations (Figure 1E).

### Mitochondrial Content Decreases Progressively During Mesoderm Differentiation

**FIGURE 2:**
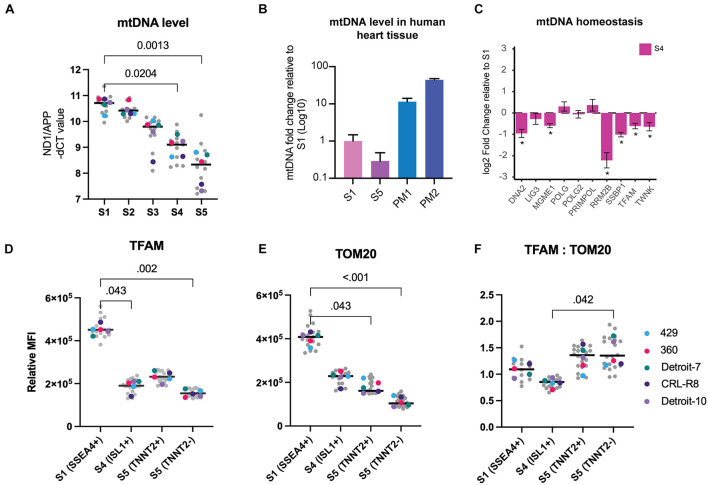
Mitochondrial mass and mtDNA decrease progressively during mesoderm differentiation. **(A)** MtDNA copy number measured during mesoderm differentiation. MtDNA level was calculated by assessing the ratio of ND1 (mtDNA gene) to APP (nuclear gene). Each data point in gray reflects a single experiment (mtDNA level of a single line from one run of differentiation at a specific stage), and each colored dot represents the median mtDNA level of each line for each stage. The black line is the median of total measurements for each specific stage. Statistical significance was assessed using a nonparametric, one-way ANOVA test followed by correction for multiple comparisons (Friedman test with Dunn’s multiple comparison test), *n* = 5. **(B)** MtDNA levels were significantly lower in both S1 and S5 cells compared with human heart tissue. The bar chart shows the average mtDNA level in cells collected from five hiPSC lines in S1 and S5 and compares this with the mtDNA levels of cells isolated from postmortem heart tissues (PM1: 59 years, female, died of lung cancer; and PM2: 65 years, male, died of colon cancer). **(C)** We used RNA-seq from dataset A (all five lines included) to assess mtDNA homeostasis and replication machinery in Isl1^+^ progenitor cells. Several genes involved in mtDNA replication exhibited significant drops in S4 relative S1. **(D)** Using TFAM as a surrogate for mtDNA, we measured TFAM levels in different cell types across three different stages of differentiation using flow cytometry, and we detected a significant loss of TFAM at stages S4 and S5 compared with S1. There was also a significant difference in TFAM levels between TNNT2^+^ and TNNT2^–^ cells. **(E)** We measured TOM20 using the same methodology and found a similar significant decrease at S4, and further reduction at S5. TNNT2^+^ cells demonstrated significantly higher levels of TOM20 relative to TNNT2^–^ cells. **(F)** The ratio of TFAM:TOM20 increased significantly from stage S4 to stage S5 of cardiac differentiation. Since TFAM binds mtDNA in stoichiometric amounts and TOM20 reflects mitochondrial content, this suggests that mtDNA levels increased per unit mitochondrial volume. In **(D–F)**, each data point in gray reflects a single experiment (MFI level of a single line from one run of differentiation at specific stage), and each colored dot represents the median MFI level of each line for each stage. The black line is the median of total measurements for each specific stage. Statistical significance was assessed using a nonparametric one-way ANOVA test followed by correction for multiple comparisons (Friedman test with Dunn’s multiple comparison test), *n* = 5. mtDNA, mitochondrial DNA; hiPSC, human induced pluripotent stem cell; MFI, median fluorescence intensity.

Thirteen polypeptides that are essential respiratory chain components are encoded by mtDNA. Unlike nuclear DNA, mtDNA is present in multiple copies, and its copy number can impact the levels of mitochondrial RNA transcripts available for generating respiratory chain subunits (Anderson et al., 1981). We assessed the mtDNA copy number at different stages of mesoderm differentiation using real-time PCR quantification relative to the nuclear gene *APP* (Tzoulis et al., 2013). This revealed a clear and progressive reduction of mtDNA copy number (≤85%) during differentiation of both hiPSCs and hESCs to the mesodermal lineage (Figure 2A and Supplementary Figure 3A). For comparative purposes, we quantified mtDNA in postmortem human heart using the same method and confirmed that the level of mtDNA in the mature tissue is at least 11–44-fold higher than in hPSCs and differentiated cells at S5 (Figure 2B).

Assessed by bulk RNA-seq, we found that the majority of genes involved in mtDNA homeostasis decreased during mesoderm differentiation, including mtDNA maintenance exonuclease 1 (*MGME1*), single-stranded DNA binding protein (*SSBP*), mitochondrial transcription factor A (*TFAM*), and mtDNA helicase (*TWNK*) (Figure 2C). We validated RNA-seq findings by using real-time PCR to quantify the expression of *SSBP* and *POLG*, which both have critical roles in mtDNA replication, and were able to show a progressive decline of *SSBP* with no significant change in *POLG* expression level (Supplementary Figures 3B–D). No evidence of qualitative damage such as mtDNA deletion was found at any stage of differentiation, which further confirms that reduction of mtDNA is not an artifact of damaged mtDNA replication machinery that can be caused, for example, by the continued culture of hPSCs (Supplementary Figures 3E,F).

The link between the TFAM protein and mtDNA has been investigated in depth (Larsson et al., 1994, 1998), and studies show that it binds mtDNA in molar quantities (Ekstrand et al., 2004; Kaufman et al., 2007; Kukat and Larsson, 2013). Using flow cytometry, we assessed TFAM both as a direct measure of a mitochondrial matrix protein and as an indirect measure of mtDNA level within each cell population co-stained with antibodies against stage-specific markers, to validate our findings from real-time PCR. We found a 58% reduction of TFAM in Isl1^+^ progenitor cells (S4) relative to the pluripotent stage (S1) (Figure 2D). The TFAM level decreased further in the TNNT2^–^ population but showed an increase in the TNNT2^+^ cells (Figure 2D).

Next, we examined changes in mitochondrial mass during mesoderm differentiation at the single-cell level. We used flow cytometry and co-staining with antibodies against stage-specific markers and Translocase of Outer Mitochondrial Membrane 20 (TOMM20), a well-established marker of mitochondrial mass. The TOMM20 level showed a significant fall (43%) from S1 to S4 and reached its lowest level at S5 (60%) (Figure 2E). As expected, the level of TOM20 was lower (∼40%) in non-cardiomyocytes than cardiomyocytes (TNNT2^+^), potentially indicating a lower level of mitochondria in non-cardiac cells. The fall in mitochondrial mass could also be followed by looking at VDAC expression. *VDAC* encodes a vital outer membrane protein that is routinely used as a mitochondrial mass marker; its expression started decreasing from S3 (Supplementary Figure 3D). Given the link between cell size and mitochondrial mass (Zheng et al., 2016), the finding of a lower mitochondrial mass was surprising; differentiated cells at S5 are much larger than PSCs (Figure 1D-iv). Interestingly, when we measured changes in mtDNA level relative to mitochondrial content, by plotting the level of TFAM (an indirect marker of mtDNA) against TOM20 (a direct marker for mitochondrial content), we found that this ratio varied during the course of mesoderm differentiation. The ratio of TFAM to TOM20 was significantly higher in differentiated cells in S5 relative to Isl1^+^ progenitor cells (S4) (Figure 2F), suggesting that the lowest level of mtDNA per unit mitochondrial mass occurred in Isl1^+^ progenitor cells and then rose in the more differentiated stage. Together, these findings suggest a progressive fall of mitochondrial content during mesoderm differentiation.

### Despite Lower Mitochondrial Content, Differentiated Cells Generate More Energy Through Tricarboxylic Acid Than Glycolysis

**FIGURE 3:**
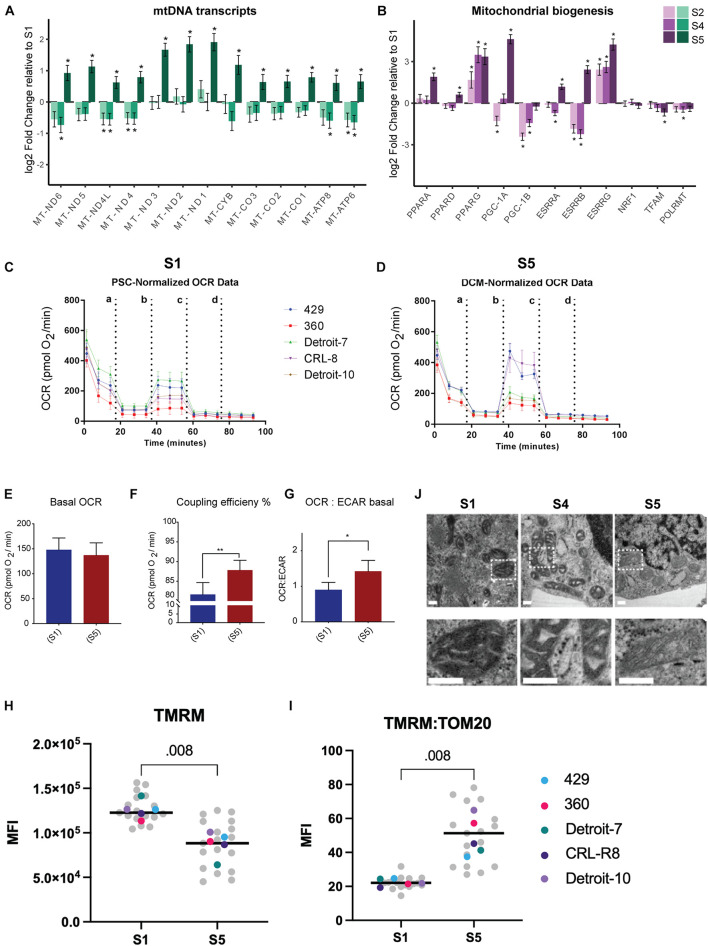
Higher mitochondrial respiration in differentiated mesodermal derivatives despite lower mitochondrial content. **(A)** The mtDNA transcripts collected via RNA-seq (Dataset B) at different stages of mesoderm differentiation suggest a significant increase in the number of mitochondrial transcripts in S5 relative to S1. **(B)** A panel of genes associated with metabolic remodeling was selected based on previous studies (Scarpulla et al., 2012; Zheng et al., 2016) to evaluate changes in gene expression at different stages of mesoderm differentiation relative to undifferentiated, stage S1 cells. Bar plots depict the estimated log twofold change ± s.e.m, *n* = 5. **(C,D)** Graphs present the result of OCR in hPSCs **(C)** and differentiated cells at S5 **(D)**. Cells were exposed sequentially to oligomycin (a), CCCP (b), rotenone (c), and antimycin A (d). **(E)** To better visualize the result of Seahorse, we presented data as bar charts. Basal OCR level did not change in differentiated cells relative to undifferentiated hPSCs, which suggests higher rates of oxygen consumption by differentiated cells since we have shown that these cells have almost 50% less mitochondria compared with hPSCs. **(F)** Coupling efficiency increased significantly in differentiated cells, which showed a higher potential for ATP generation relative to undifferentiated hPSCs. **(G)** The basal OCR:ECAR ratio showed an increase in differentiated cells, confirming that these cells are more reliant on OXPHOS compared with undifferentiated hPSCs. Each bar in **(E–G)** shows mean ± SD, *n* = 5; and two-tailed unpaired *t*-tests with Welch’s correction were chosen to assess the statistical significance of the difference between undifferentiated hPSCs and S5. In all bar charts, * *p* < 0.05 and ** *p* < 0.01. **(H)** We assessed mitochondrial membrane potential by treating live cells with TMRM and subsequently with FCCP and plotting the difference in delta MFI (TMRM-MFI - FCCP-MFI) for each stage. We found an apparent higher TMRM concentration in hPSCs relative to S5 cells. **(I)** Next, we used the TOM20-MFI collected from a previous step (Figure 2E) and computed TOM20-MFI for all live cells in S1 and S5 (both TNNT2^+^ and TNNT2^–^ included) as an indicator of mitochondrial mass. We then adjusted the TMRM level to the mitochondrial content of cells in each stage (TMRM-MFI:TOM20-MFI). The ratio of TMRM and TOM20 suggested a higher level of mitochondrial membrane potential per unit of mitochondrial mass for differentiated cells compared with undifferentiated hPSCs. In H and I plots, each data point in gray reflects a single experiment (MFI level of a single line from one run of differentiation at specific stage), and each colored dot represents the median MFI level of each line for each stage. The black line is the median of total measurements for each specific stage. Statistical significance was assessed using a two-tailed unpaired nonparametric *t*-test (Mann–Whitney test), *n* = 5. **(J)** Representative TEM pictures of mitochondria during cardiomyocyte differentiation of an hiPSC line (Detroit-7) at S1, S4, and S5. Mitochondria appear smaller with wider, more immature cristae at stages S1 and S4; while at S5, more mitochondria show an expanded matrix with typical narrow, mature cristae. The bottom panel is the magnification of the indicated areas in the top panel. Scale bar: 200 nm. MtDNA, mitochondrial DNA; OCR, oxygen consumption rate; hPSC, human pluripotent stem cell; OXPHOS, oxidative phosphorylation; MFI, median fluorescence intensity; TEM, transmission electron microscope; hiPSC, human induced pluripotent stem cell.

Despite analyzing a heterogeneous population (TNNT2^+^ and TNNT2^–^ cells), we were able to verify a significant increased expression of mtDNA genes in S5 relative to other stages of differentiation via bulk RNA-seq (Figure 3A). Demonstrating a significant increase in the expression of mtDNA genes in the differentiated cells in S5 suggests that differentiated cells rely more on mitochondrial respiration, regardless of the cell type they are committed to, and despite having much lower mitochondrial mass. Thus, we assessed mitochondrial oxidative activity by measuring OCR in undifferentiated stem cells (Figure 3C) and differentiated cells at S5, which contain beating cardiomyocytes (see Supplementary Movies 3, 4 and Figure 3D), using a Seahorse XF^e^96 extracellular flux analyzer. Interestingly, despite a major drop in mitochondrial mass, no major change in basal respiration—the amount of oxygen consumed by mitochondria under basal conditions—was detected: differentiated cells at S5 137 pmol/min, undifferentiated hPSCs at S1 148 pmol/min (Figure 3E and Supplementary Figure 4A). This suggests that the remaining mitochondria have higher levels of oxygen consumption that compensates for the lower mitochondrial mass in differentiated cells. Maximal OCR and spare capacity (also known as reserve capacity) showed a slight increase in S5 compared with S1 cells (Supplementary Figures 4B,C), while the coupling efficiency—the proportion of mitochondrial respiratory chain activity used to drive ATP synthesis (Divakaruni and Brand, 2011)—was significantly higher (Figure 3F). The ratio of OCR to extracellular acidification rate (ECAR), an indicator of how much lactate is produced through glycolysis, also rose in S5 cells relative to S1, suggesting a shift to mitochondrial respiration (Figure 3G). The finding of a negative value for spare capacity in hPSC lines (360 and CRL-8) raised the possibility that uncoupling by CCCP had collapsed the membrane potential in these lines (see blue bars in Supplementary Figure 4A-iv) and that, with a respiratory chain already working maximally, there was no further reserve capacity to be used with the extra stress (Varum et al., 2011; Zhang et al., 2012b).

We also investigated mitochondrial membrane potential as a marker for mitochondrial activity. Membrane potential is generated by proton movement driven by the electron transport chain; and although mitochondrial membrane potential is an excellent marker for assessing mitochondrial function, it also reflects mitochondrial volume (Perry et al., 2011). We therefore employed a lipophilic cationic fluorescent probe, TMRM, to evaluate mitochondrial membrane potential at the single-cell level by flow cytometry; and we normalized our data to the MFI of TOM20 from the same differentiation batch to further adjust membrane potential to mitochondrial content within a single cell (see Supplementary Methods 1.10). We did not use cell stage-specific markers for this assessment, as this was done in live cells; and the result therefore reflects the membrane potential of total live cells in S1 and S5. The absolute median MFI of TMRM was significantly lower at S5 relative to S1 (Figure 3H and Supplementary Figure 4E); however, when we adjusted the MFI of TMRM for mitochondrial mass, using the ratio of MFI-TMRM to MFI-TOM20, we found a more than twofold increase in mitochondrial membrane potential at S5 compared with undifferentiated hPSCs at S1 (Figure 3I and Supplementary Figure 4F). The significantly higher mitochondrial membrane potential per unit of mitochondrial mass in differentiated cells at S5 relative to undifferentiated hPSCs further suggested that the mitochondria in differentiated cells are more efficient in generating ATP.

### Crista Remodeling in Differentiated Cells

We examined cells collected on S1, S4, and S5 by TEM to assess the mitochondrial morphology and crista structure. The mitochondria in S1 appear to have wide cristae with a dense matrix, while in S4 and S5, cristae appear more compact with a clearer matrix (Figure 3J). We also identified a significant increase in the expression of genes associated with mitochondrial biogenesis and respiration activities (e.g., *PPARA*, *PPARG*, *PGC-1A*, and *ESSRA*; see Figure 3B and Supplementary Figure 5). Since no increase in mitochondrial mass was demonstrated relative to Isl1^+^ progenitor cells (Figure 2E), together, these data suggested remodeling of mitochondrial membrane and cristae as a potential explanation for the increase in mitochondrial respiration.

## Discussion

The control of mitochondrial biogenesis and ATP generation is a dynamic and complex process that is involved in governing pluripotency and pathways of differentiation (Friedman and Nunnari, 2014; Wanet et al., 2015). Previous studies have shown that following the inactivation of mtDNA replication during preimplantation, mtDNA levels fall as copies segregate into the newly divided daughter cells, reaching their lowest level at the embryoblast stage before starting to increase at some point during differentiation (Pikó and Taylor, 1987; Cao et al., 2007). Although the first part of this process is well established (Taylor and Pikó, 1995; Floros et al., 2018), the timing of the second part of the process remains uncertain. Earlier studies suffer from low sample number and the tendency to focus only on the comparison between differentiated and undifferentiated cells without considering transitional states during differentiation or the heterogeneous nature of the cultures resulting from spontaneous differentiation (St John et al., 2005; Cho et al., 2006). Importantly, recent findings suggest that metabolic switching during differentiation is germ layer-specific and is regulated differently in the ectoderm compared with mesoderm and endoderm (Cliff et al., 2017).

Our findings corroborate previous studies showing a change in energy profile between hPSCs and terminally differentiated cells with a shift from glycolysis to OXPHOS (Folmes et al., 2012). However, contrary to what was previously thought, we show that mitochondrial content drops progressively at the start of differentiation in mesoderm, a trend that continues despite increased mitochondrial activity and higher levels of ATP-linked respiration. A reduction of both mtDNA and mitochondrial mass was detected in the very early stages of ectoderm differentiation, but both markers started to increase again from the neuroprogenitor cell stage onward (Birket et al., 2011; Zheng et al., 2016; Lees et al., 2018). A decline in mtDNA content has also been reported during hematopoietic differentiation, as well as during differentiation of hESCs toward primordial germ cells (de Almeida et al., 2017; Floros et al., 2018). Thus, our findings in a mesodermal lineage suggest that the reduction of mtDNA and mitochondrial content from pluripotent to multipotent stem cells may be a more general phenomenon than previously thought and not limited to one specific cell lineage. At the same time, our results showing that this loss continues up to and including differentiated cells at S5 suggest that there are cell-lineage-specific metabolic pathways following commitment to a specific cell type. This contrasts with earlier studies that suggested a simultaneous expansion of mitochondrial content and increase in OXPHOS activity during all lineage differentiation.

Our findings showing that the lowest level of mtDNA per unit mitochondrial mass was observed in cardiac progenitor cells are very similar to those reported in neural progenitor cells during ectoderm differentiation (Lees et al., 2018). A possible explanation for the reduction in mitochondrial content during very early stages of differentiation is that this occurs in order to generate a mitochondrial bottleneck to select mtDNA with specific variants and specialized mitochondria for development of specific germ layers (Gong et al., 2015; Floros et al., 2018; Rossmann et al., 2021). MtDNA bottlenecks were originally thought to be limited to the germline; however, evidence increasingly suggests the presence of cell-specific mtDNA bottlenecks during development (Zhang et al., 2018). This phenomenon may, in part, explain the observed differences between mtDNA levels in differentiated cells in S5 and postmortem human heart tissue. Another important factor in our protocol, which gave rise to low numbers of cardiomyocytes, is that the resulting functionally differentiated cardiomyocytes poorly reflect characteristics of mature cardiomyocytes *in vivo*. For example, it is known that hiPSC-derived cardiomyocytes are transcriptionally, structurally, and functionally immature and resemble fetal cardiomyocytes (for review, see Karbassi et al., 2020). Other factors include differences in cell-type composition (e.g., absence of interaction between cardiac progenitors and surrounding tissue; Miquerol and Kelly, 2012), maturity level, and microenvironment (e.g., presence of fatty acids as a source of energy), which are not mutually exclusive. Epigenetic modifications add another layer of complexity to stage-specific mtDNA regulation, as was shown by in-depth profiling of human heart tissue during development (Gilsbach et al., 2018).

In this study, we assessed cells starting 48 h after WNT activation, when expression of mesendoderm markers (e.g., MESP1) was stabilized, while other studies demonstrated a metabolic shift starting as early as 24 h after CHIR99021 treatment (Cliff et al., 2017). One of the limitations of our study is that we have not investigated this initial timepoint, which is particularly important in mesoderm differentiation, as a very recent study suggested that assessing metabolic switch during the first 24-h time window can be used as an indicator for efficiency of cardiomyocyte differentiation (Qian et al., 2021). More importantly, our findings demonstrating variation in the mtDNA level relative to mitochondrial mass highlight that mtDNA levels may not act as a proxy for mitochondrial level, particularly during differentiation, and that it is therefore important to use a complementary method to assess mitochondrial levels directly. Our findings also emphasize the importance of differentiating between active loss of mtDNA and failure to expand mtDNA copy number when studying tissues derived from mesodermal lineages in infants with mitochondrial disease.

Collectively, high levels of oxygen consumption, lactate reduction, and significantly higher level of mitochondrial membrane potential at S5 relative to undifferentiated cells demonstrate the metabolic shift from glycolysis to OXPHOS, despite lower mitochondrial content. Together, our findings suggest that the mitochondria in terminally differentiated cells are indeed more mature and more efficient at generating ATP. These findings offer experimental validation for the previously proposed mathematical model that suggests mammalian cells can modulate their mitochondrial membrane potential, rather than their mtDNA level, to quickly adapt to changes in energy demands (Miettinen and Björklund, 2016; Aryaman et al., 2017). Previous findings have revealed that mitochondrial inner membrane morphology modulates OXPHOS function by modifying the kinetics of chemical reactions and regulating the arrangement of protein complexes (Cogliati et al., 2016). It is also known that modulating crista structure affects mitochondrial respiratory efficiency independently of changes to mitochondrial protein synthesis (Cogliati et al., 2013). Our findings align with previous studies that reported the formation of cristae and mitochondrial permeability transition pore (mPTP) closure in differentiated cardiomyocytes, which are known characteristics of the developmental maturation of mitochondria (Hom et al., 2011; Teixeira et al., 2015; Dai et al., 2017).

Whether our findings are physiologically relevant or reflect changes in signaling pathways during the early stages of differentiation under the normoxic culture conditions remains an unanswered question. Nevertheless, our findings emphasize the importance of investigating changes in mitochondrial properties across different cell lineages, and highlight a narrow time window during differentiation that is crucial in the relationship between mitochondrial remodeling and cell fate that can be a target for future investigations. These findings may have significant implications in developing strategies for lineage-directed differentiation. Better understanding of the molecular basis of mitochondrial remodeling during differentiation can potentially help us to better understand the key regulatory mechanism underlying the pathophysiology of mitochondrial and degenerative diseases and will further assist us in identification of novel therapeutic agents targeting metabolic pathways. We suggest that future studies use more advanced iPSC-based models [e.g., cardiac spheroids (Giacomelli et al., 2017) and gastruloids (Rossi et al., 2021)] that more closely mimic early human cardiogenesis in combination with single-cell transcriptomic analysis to gain even deeper understanding of the underlying molecular mechanisms.

## Data Availability Statement

The original contributions presented in the study are publicly available. This data can be found here: https://www.ebi.ac.uk/ena/browser/view/PRJEB47044?show=reads.

## Author Contributions

LB and NB: conceptualization and supervision. NB, SM, and GS: methodology. GN: software. NB, SM, and GN: validation and formal analysis, and visualization. SM, NB, IP, and RS: investigation. LB, GS, and CT: resources. GN and NB: data curation. NB: writing—original draft preparation. LB, NB, and SM: writing—review and editing. LB: project administration and funding acquisition.

## Conflict of Interest

The authors declare that the research was conducted in the absence of any commercial or financial relationships that could be construed as a potential conflict of interest.

## Publisher’s Note

All claims expressed in this article are solely those of the authors and do not necessarily represent those of their affiliated organizations, or those of the publisher, the editors and the reviewers. Any product that may be evaluated in this article, or claim that may be made by its manufacturer, is not guaranteed or endorsed by the publisher.

## Footnotes

1
https://git.app.uib.no/gni042/cardiomyocites-rna-seq
