# Supplementary material for: Distinct Mitochondrial Remodeling During Mesoderm Differentiation in a Human-Based Stem Cell Model
Source: Front Cell Dev Biol. 2021 Oct 14;9:744777. doi: 10.3389/fcell.2021.744777 (PMC8553110; doi:10.3389/fcell.2021.744777)
Supplement: Supplementary file 1 [file Data_Sheet_1.PDF]

Supplementary Material

1 Supplementary Methods

1.1 Cardiomyocyte differentiation

Cardiomyocyte differentiation was performed in 96-well microplate as previously described(Balafkan et al., 2020). HiPSCs were seeded at 2.4 x 10^4 cells/cm and propagated on Geltrex (#A1413302, Thermo Fisher Scientific) and diluted (1:100) in Advanced DMEM/F-12 (#12634010, Thermo Fisher Scientific) under feeder-free conditions in Essential 8 Medium (E8) (#A1517001, Thermo Fisher Scientific). Within three days, when cells reached the optimum confluency, 60% to 70%, cardiomyocyte differentiation was started by applying the GSK3 inhibitor CHIR99021 (#4423, Tocris Bioscience) in RPMI 1640 (#61870, Thermo Fisher Scientific) medium supplemented with B27-without insulin (RPMI-B27) (#A1895601, Thermo Fisher Scientific) in a concentration-cell-dependent manner. After 24 hours, the medium was changed to RPMI-B27 without CHIR99021. The differentiation process was continued by adding 5 µM inhibitor of WNT production-2, IWP2 (#3533, Tocris Bioscience), diluted in RPMI-B27, 72 h post-differentiation induction for 48 h. Fresh RPMI-B27 medium was provided on day five, and from day seven cells were fed with fresh RPMI medium supplemented with B27 with insulin (#17504044, Thermo Fisher Scientific) without extra supplement) every two days.

1.2 Gene expression analysis

MagMAX™-96 Total RNA Isolation Kit (#AM1830, Thermo Fisher Scientific) was used for RNA isolation from cultured cells. Cells were rinsed with DPBS (#14190250, ThermoFisher) and lysis buffer, provided in the kit, was added directly to the cells. Lysates were immediately used for RNA isolation using automated MagMAX express 96 or stored at -80°C. EXPRESS One-Step Superscript qRT-PCR Kit (#11781-01K, Thermo Fisher Scientific) was used for cDNA synthesis from 70ng of RNA (> 30ng/µl), and real-time PCR using TaqMan probes (Table S1) on an Applied Biosystems 7500-Fast real-time PCR System (Thermo Fisher Scientific). All real-time PCR reactions were performed in triplicate and the average Ct values were normalized to the geometric mean of *ACTB* and *GAPDH* as endogenous control genes. The result (dCt) was used for further analysis. A stable expression of housekeeping genes (i.e., *ACTB* and *GAPDH*) was confirmed prior to use in Realtime PCR. In our hands, expression of *RPLPO*—another well-known housekeeping gene—was not constant during mesoderm differentiation.

1.3 RNA sequencing

RNA sequencing was carried out on two different data sets, that were analyzed independently and separately. Dataset A contained RNA sequences from hESCs and hiPSC lines collected from S1(Undifferentiated cells, day 0) and S4 (cardiac progenitors, day 7) of cardiomyocyte differentiation. Dataset A was composed of samples collected from three independent differentiations of two hESC lines (429 and 360), two independent differentiations of Detroit-7 and CRL-8, and one differentiation of Detroit-10. Dataset B corresponded to RNA sequences collected from four stages ranging from S1 to S5 (days 0, 2, 5 and 15) of three independent differentiation runs of the H1 line (hESC). The total RNA samples were isolated using MagMAX™-96 Total RNA Isolation Kit. Sample information is shown in Supplementary Tables 3 and 4.
RNA sequencing for the dataset A was carried out at the Finnish Microarray and Sequencing Centre's analysis service and Biocenter Finland. The quality of the total RNA samples (> 30 ng/µl) and libraries was ensured with Advanced Analytical Fragment Analyzer. Sample

concentration was measured with Qubit® Fluorometric Quantitation, Life Technologies.
RNA quality, as measured by RNA integrity number (RIN), was well above seven for all samples (median RIN = 9,25)—with the exception of a single sample, with RIN = 5.5. The samples were sequenced with an Illumina HiSeq 3000 instrument. We performed single-read sequencing with 1 x 50bp read length, followed by a dual index 8bp run. The base calling was performed using Illumina's standard bcl2fastq2 software, automatic adapter trimming was used. RNA sequencing for dataset B was carried out at the HudsonAlpha Genome Sequencing Center, USA, at 100bp paired-end reads. RNA quality as measured by RIN was well above seven for all samples (median RIN = 8,8). Raw fastq files were deposited in the European Nucleotide Archive (ENA) with accession number PRJEB47044. RNA sequencing analyses were performed independently for each of the datasets. Raw FASTQ files were assessed using fastQC version 0.11.8 (Andrews, 2010), and reads were mapped using Salmon version 1.3.0 (Patro et al., 2017) with the fragment-level GC bias correction option (-gcBias) against the GENCODE Release 32 (GRCh38.p13) reference transcriptome and the GRCh38 reference genome, including the whole genome as decoy sequences. Transcript quantification was collapsed to the gene-level using the R package tximport version 1.14.2 (Soneson et al., 2015) with default parameters and the GENCODE Release 32 (GRCh38.p13) annotation. Low-expressed genes (i.e., genes with less than ten reads in more than 75% of the samples of the corresponding dataset) were filtered out, resulting in 19,273 genes in dataset A—of which, 80% annotated as protein-coding— and 22,480 in dataset B (73% annotated as protein-coding).

1.4 Differential gene expression and functional enrichment analyses

Differential gene expression analyses were performed using the DESeq2 R package version 1.26 (Love et al., 2014) with default parameters. Samples from dataset A originated from different cell lines, and this was accounted for explicitly in the model by incorporating it as a covariate into the statistical model. Multiple hypothesis testing was performed with the default automatic filtering of DESeq2 followed by false discovery rate (FDR) calculation by the Benjamini-Hochberg procedure. Analyses were carried out independently for the two datasets. Genes were scored according to their significance by transforming the p-values to account for direction of change. In summary, for each gene, the up-regulated score (S_{up}) was calculated as $S_{up} = 1 – p/2$ if LFC < 0, and $p/2$ if LFC≥0. The down-regulated score (S_{down}) as $S_{down} = 1–S_{up}$, where LFC corresponds to the log fold change and p to the nominal p-value of the gene. Genes were then tested for enrichment using alternatively log(S_{up}) and log(S_{down}) scores employing the gene score resampling method implemented in the ermineR package version 1.0.1, an R wrapper package for ermineJ (Gillis et al., 2010) with the complete Gene Ontology (GO) database annotation (Ashburner et al., 2000), the KEGG database (Kanehisa and Goto, 2000) and a curated version of mitocarta (Calvo et al., 2016; Gaare et al., 2018) to obtain lists of up- and down-regulated pathways for each cohort. The source code for the RNA sequencing analyses is available in the GitLab repository (https://git.app.uib.no/gni042/cardiomyocites-rna-seq) under the GPL public license v3.0.

1.5 Flow cytometry

Cells were washed in DPBS (#14190250, ThermoFisher) three times and dissociated into single cell suspension using TrypLE™ Express Enzyme (#12604013, Thermo Fisher Scientific) for 10–20 minutes at 37°C, and were collected in the micro centrifuge tube. After a quick wash with DPBS cells were stained with Zombie Red™ Fixable Viability Kit (#423110, BioLegend) according to the manufacturer's instructions. Single cells were fixed with 4% (vol/vol) paraformaldehyde for ten minutes at room temperature (RT) and permeabilized for ten minutes at -20°C with 90% ice-cold methanol diluted in PBS. Cells were blocked in blocking buffer consisting of 0.3M glycine, 5% goat serum, and 1% BSA in PBS for 20 minutes at RT. We used the optimal concentration of conjugated antibodies for flow cytometry reported by the supplier for

each batch. Antibodies are listed in Supplementary Table (Table S1). All the primary and secondary antibodies, as well as conjugated antibodies, were diluted in blocking buffer and stained for 30 minutes at RT. Cells were washed after staining and collected in 300µl of 1% FBS in PBS. Samples were analyzed quickly after staining. At least 30.000 events were collected for the target marker using a Sony cell sorter SH800 (Sony Biotechnology Inc.), and data was analyzed and presented by FlowJo V.10.5.0 (FlowJo LLC, OR, USA, www.FlowJo.com). 8-Peaks Rainbow Calibration Particles (Biolegend #422903) was used for routine alignment and performance verification of the flow cytometry. The flow cytometer was calibrated prior to quantitative fluorescence intensity measurements using Quantum™ Alexa Fluor® 488 MESF (molecules of equivalent soluble fluorophore) (Bangs Laboratories, Inc. #488). In order to test the integrity of the results over time, collected median fluorescence intensity (MFI) was normalized to MESF as an external control and the resulting values reported as *relative MFI*. All gates were adjusted according to fluorescence minus one control (FMO). To analyze the flow cytometry data the median values of relative MFI were used to compare the samples.

1.6 Immunocytochemistry and fluorescence microscopy

Cells were seeded on Geltrex coated cover slips or in Millicell® EZ SLIDES (#PEZGS0816, Merck Millipore). Cells were fixed with 4% (vol/vol) paraformaldehyde for ten minutes at RT and permeabilized with 0.3% Tween 20 (#822184, Merck Millipore). All the primary and secondary antibodies were diluted in blocking buffer consisting of 0.3M glycine, 5% goat serum, and 1% BSA in PBS. A final concentration of 10µg/ml was used for all primary antibodies and they were incubated overnight at 4°C. Alexa Fluor 488 or 594-conjugated (Thermo Fisher Scientific) secondary antibodies were diluted in blocking buffer 1:1000 and incubated for 30 minutes to 1hour at RT. Nuclei were stained with Gold Antifade Reagent with DAPI (#P36935, Thermo Fisher Scientific). Confocal microscopy images were taken on a Zeiss LSM 510 META or a Leica TCS SP5 at the Molecular Imaging Center (MIC), University of Bergen, and data analysis and image editing were done with Fiji (Schindelin et al., 2012). Antibodies are listed in Supplementary Table (Table S1).

1.7 Transmission electron microscopy

Cells were washed in DPBS and dissociated into single cell suspension using TrypLE™ Express Enzyme for 10–20 minutes at 37°C, and were collected in the micro centrifuge tube and centrifuged at 300g for ten minutes at RT. Cells were fixed in 2,5% glutaraldehyde (diluted in a 0,1M sodium cacodylate buffer) for 24 hours at 4°C and delivered to MIC facility at the University of Bergen. Post-fixation was performed for 1hour (on ice) in 1% osmium tetroxide (EMS # 19134) diluted in 0,1M sodium cacodylate buffer, followed by two washing steps. The samples were then dehydrated using a graded ethanol series (30%, 50%, 70%, 96% and 100%) before being transferred to a 1:1 solution of 100% ethanol:propylene oxide (15 minutes). Samples were then transferred to 100% propylene oxide (15 minutes) before gradually introducing agar 100 resin (AgarScientific R1031) drop-by-drop over the next hours. Samples were then transferred to a small drop of 100% resin, and excess propylenoxid was allowed to evaporate (1hour). Samples were then transferred to 100% resin and placed in molds and left at RT overnight. The molds were placed at 60°C for 48 hours to polymerize. Ultrasections of approximately 60nm were placed on 100 mesh formvar coated (EMD # 15820) copper grids (EMS #G100H-Cu) and stained with 2% uranyl acetate (EMS # 22400) and lead citrate (VWR #1.07398). Grids were imaged using a Jeol JEM-1230 transmission electron microscope at 80kV.

1.8 MtDNA analysis

MtDNA quantification and deletion assessment was performed in DNA isolated from cultured cells using MagMAX™-96 DNA Multi-Sample Kit (#4413021, Thermo Fisher Scientific) and

real-time PCR, as well as long range PCR, as previously described (Tzoulis et al., 2013). A commonly deleted region (MT-ND4) in the major arc of mitochondrial DNA, and a rarely deleted region (MT-ND1), were utilized to quantify deletion. MT-ND1 was compared with amplification of a single-copy nuclear gene (APP) to assess the number of mtDNA copies. A triplex reaction of ND1, ND4 and APP was performed simultaneously within the same well using a 7500 fast sequence detection system (Thermo Fisher Scientific). Efficiency of the triplex reaction was measured prior sample analysis.

1.9 Measurement of OCR and ECAR using Seahorse XF-96 analyzer

Respiration and acidification rates were measured on monolayer culture of undifferentiated hPSCs and cells at S5 using a Seahorse XFe96 extracellular flux analyzer (Agilent, Santa Clara, CA, US). Geltrex coated XFe96 assay plates were utilized for seeding of $3x10^3$ undifferentiated hPSCs and $2x10^5$ hPSC-derived cardiac cells in each well. Cells were cultured in cell specific normal growth medium supplemented with 10µM of Y-27632 for 24 hours at 37°C and 5% CO_2. The next day, the supplemented growth medium was replaced with normal growth medium without supplement and kept until they reached almost 90% confluency prior to analysis. In order to find the optimal concentration of Carbonyl cyanide m-chlorophenyl hydrazone (CCCP) and oligomycin for each cell type, they were titrated prior to the cell analysis. The assay was performed in assay medium (pH 7.4, unbuffered) that was supplemented with 2mM L-glutamine, 2mM sodium pyruvate and 10mM glucose. Cells were washed twice and pre-incubated in the assay medium and kept in a CO_2-free incubator (XF Prep Station, Seahorse Biosciences) at 37°C for 1 hour before measurement, in order to remove CO_2 from the medium. For mitochondrial respiration analysis, the final concentrations of 3µM oligomycin, 0.5-1µM CCCP, 1µM rotenone, and 1µM antimycin A were diluted in assay medium. In order to correct the final results for differences in cell size and number between the undifferentiated hPSCs and differentiated cells at S5, we measured the total protein concentration for each well using absorbance at 280nm. All the results were reported as pmol O_2 per min after normalized to their total protein concentration. Each parameter was calculated accordingly; basal OCR= (OCR in non-treated cell–OCR after adding Antimycin A), Coupling Efficiency = (ATP Production Rate / Basal Respiration Rate × 100), spare respiration capacity = (maximal respiration / basal respiration). The XF reader software (Wave Desktop 2.4) was used to analyses the data.

1.10 Measurement of mitochondrial membrane potential

We used a previously described protocol (Rowe and Boletta, 2013) to analyze the mitochondrial membrane potential (ψm) independent of cell volume using TMRM. We quantified the fluorescent intensity of TMRM (FI) before and after applying Carbonyl cyanide 4-(trifluoromethoxy)phenylhydrazone (FCCP) as an uncoupler for OXPHOS which eliminates the mitochondrial membrane potential. The distribution of TMRM relies on the Nernstian equilibrium(Dykens and Stout, 2001). Accumulation of TMRM in the cytosol is very small compared with mitochondria due to the low negative charge of cytosol relative to mitochondrial matrix. Thus, fluorescence readouts mirror the mitochondrial matrix:cell volume ratio. FCCP treatment of the cell releases the TMRM from mitochondrial matrix into cytosol and causes a re-equilibrium of TMRM in the cytosol. Therefore, fluorescence readout after FCCP treatment represents cell volume. The difference between median for TMRM and TMRM-FCCP treated cells provided us the membrane potential level normalized to the cell size. We analyzed mitochondrial membrane potential by flow cytometry starting with $1x10^6$ cells per sample. Each sample was divided in two; one sample was treated with 25nM TMRM and another with a mixture of 25nM TMRM and 100µM FCCP. Samples were incubated for 20 minutes at 37 °C and 5% CO_2, washed three times with HBSS, and analyzed within 1 hour. In order to adjust the measured membrane potential to the mitochondrial content of cells at single cell level, we used median of TMO20-MFI for live cells at S1 and S5 as an indicator of mitochondria mass. Using

the ratio of TMRM-MFI in live cells (only viable cells can take up TMRM) at S1 and S5 to TOM20-MFI of the total live cells (TNNT2+ and – are included for S5) from the same batch of differentiation we calculate the mitochondrial membrane potential per unit of mitochondrial mass (for more details see (Perry et al., 2011)).

1.11 References

Andrews, S. (2010). FastQC: a quality control tool for high throughput sequence data. Available at: https://www.bioinformatics.babraham.ac.uk/projects/fastqc/RNA-Seq_fastqc.html.

Ashburner, M., Ball, C. A., Blake, J. A., Botstein, D., Butler, H., Cherry, J. M., et al. (2000). Gene ontology: tool for the unification of biology. The Gene Ontology Consortium. *Nat Genet* 25, 25–9. doi:10.1038/75556.

Balafkan, N., Mostafavi, S., Schubert, M., Siller, R., Liang, K. X., Sullivan, G., et al. (2020). A method for differentiating human induced pluripotent stem cells toward functional cardiomyocytes in 96-well microplates. *Sci Rep-uk* 10, 18498. doi:10.1038/s41598-020-73656-2.

Calvo, S. E., Clauser, K. R., and Mootha, V. K. (2016). MitoCarta2.0: an updated inventory of mammalian mitochondrial proteins. *Nucleic Acids Res* 44, D1251-7. doi:10.1093/nar/gkv1003.

Dykens, J. A., and Stout, A. K. (2001). Assessment of mitochondrial membrane potential in situ using single potentiometric dyes and a novel fluorescence resonance energy transfer technique. 65, 285–309. doi:Doi 10.1016/S0091-679x(01)65018-0.

Gaare, J. J., Nido, G. S., Sztromwasser, P., Knappskog, P. M., Dahl, O., Lund-Johansen, M., et al. (2018). Rare genetic variation in mitochondrial pathways influences the risk for Parkinson's disease. *Movement Disord* 33, 1591–1600. doi:10.1002/mds.64.

Gillis, J., Mistry, M., and Pavlidis, P. (2010). Gene function analysis in complex data sets using ErmineJ. *Nat Protoc* 5, 1148–59. doi:10.1038/nprot.2010.78.

Kanehisa, M., and Goto, S. (2000). KEGG: kyoto encyclopedia of genes and genomes. *Nucleic Acids Res* 28, 27–30. doi:10.1093/nar/28.1.27.

Love, M. I., Huber, W., and Anders, S. (2014). Moderated estimation of fold change and dispersion for RNA-seq data with DESeq2. *Genome Biol* 15, 550. doi:10.1186/s13059-014-0550-8.

Patro, R., Duggal, G., Love, M. I., Irizarry, R. A., and Kingsford, C. (2017). Salmon provides fast and bias-aware quantification of transcript expression. *Nat Methods* 14, 417–419. doi:10.1038/nmeth.4197.

Perry, S. W., Norman, J. P., Barbieri, J., Brown, E. B., and Gelbard, H. A. (2011). Mitochondrial membrane potential probes and the proton gradient: a practical usage guide. *Biotechniques* 50, 98–115. doi:10.2144/000113610.

Rowe, I., and Boletta, A. (2013). Mitochondrial Transmembrane Potential (ψm) Assay Using TMRM. *Bio-protocol* 3. doi:10.21769/bioprotoc.987.

Schindelin, J., Arganda-Carreras, I., Frise, E., Kaynig, V., Longair, M., Pietzsch, T., et al. (2012). Fiji: an open-source platform for biological-image analysis. *Nat Methods* 9, 676–82. doi:10.1038/nmeth.2019.

Soneson, C., Love, M. I., and Robinson, M. D. (2015). Differential analyses for RNA-seq: transcript-level estimates improve gene-level inferences. *F1000research* 4, 1521. doi:10.12688/f1000research.7563.2.

Supplementary Figure 1

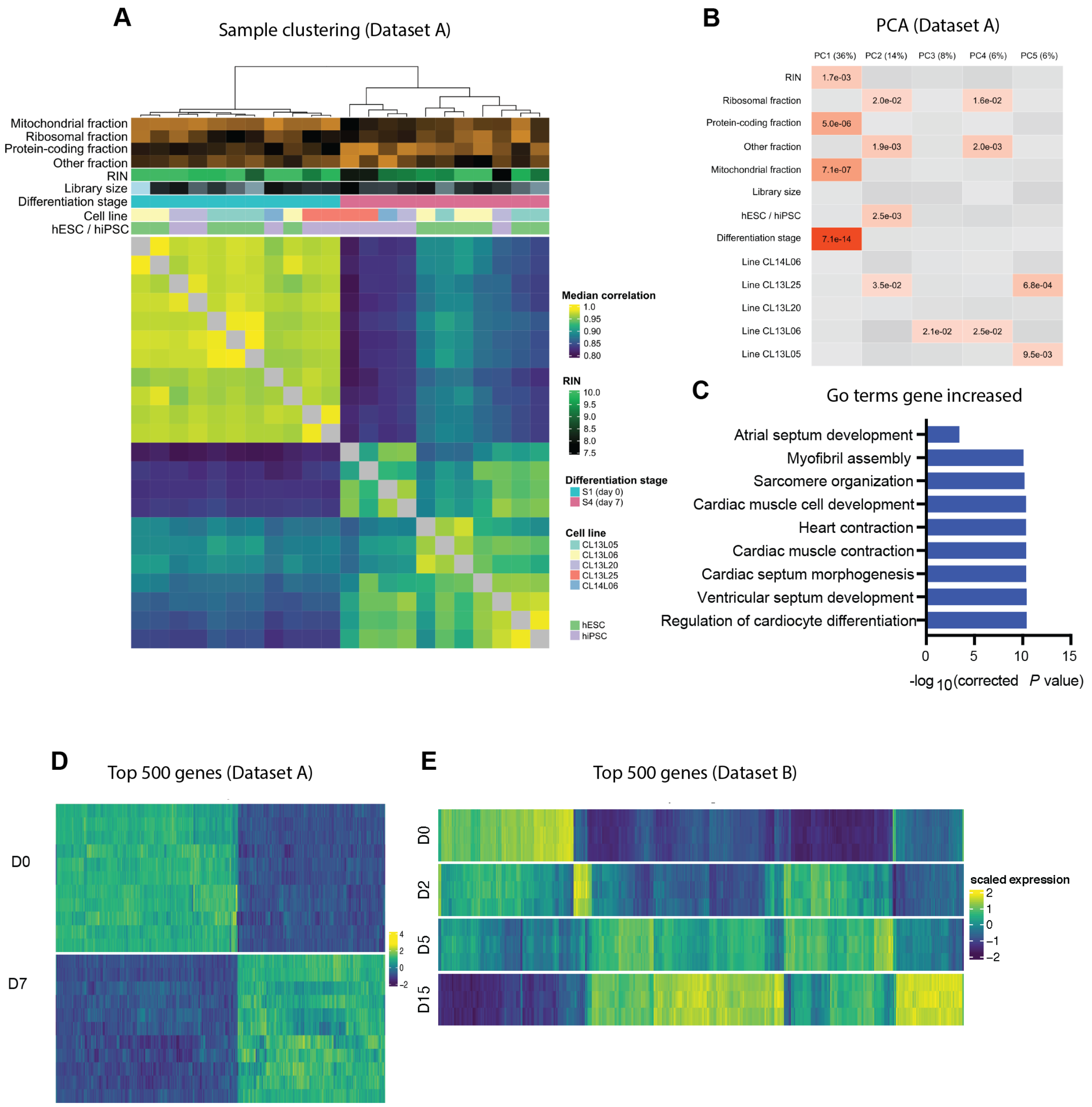

Supplementary Figure 1. (A) Sample clustering from Dataset A (stages S1 and S4) based on normalized RNA-seq counts. The heatmap depicts median sample correlation in expression, with column annotation based on experimental variables. **(B)** Association between main lines of variation in gene expression (first five principal components) and experimental variates for Dataset A (stages S1 and S4). P values below 0.05 are annotated in the cells (linear regression model). **(C)** GO term enrichment analysis shows up-regulated cellular pathways during cardiomyocyte differentiation (Dataset B). Barplot representing -log10 corrected p-values for gene set enrichment analysis of representative pathways involved in cardio-myocyte differentiation, all up-regulated. **(D)** Heatmap representing scaled gene expression for the top 500 differentially expressed genes (columns) between day 0 and day 7 for all samples in dataset A (rows). **(D-E)** Heatmaps representing scaled gene expression for the top 500 differentially expressed genes (columns) across time for all samples in each dataset (rows).

Supplementary Figure 2

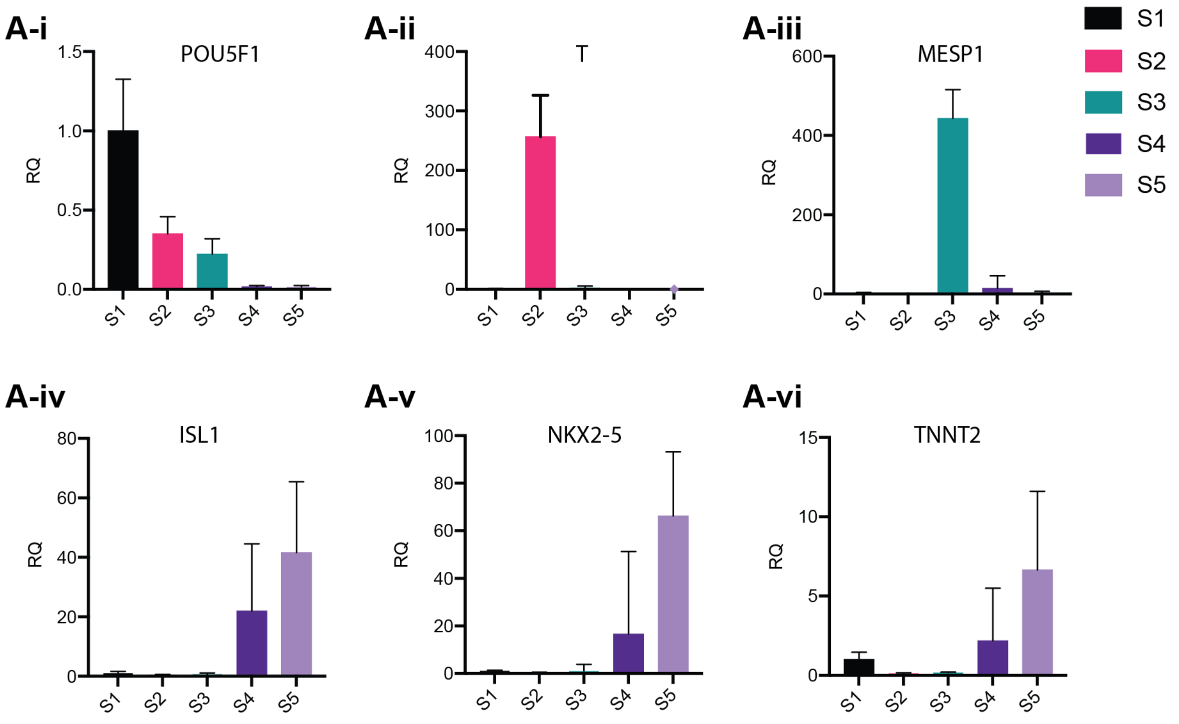

Supplementary Figure 2. Transcriptomic profiling of cells during cardiomyocyte differentiation. (A i-vi) Gene expression of selected markers is individually examined using real-time PCR for different stages of cardiomyocyte differentiation. Markers include: POU Class 5 Homeobox 1 (POU5F1), T-Box Transcription Factor T (T), Mesoderm Posterior BHLH Transcription Factor 1 (MESP1), ISL LIM Homeobox 1 (ISL1), NK2 Homeobox 5 (NKX2-5), and Cardiac Muscle Troponin T (TNNT2). HESC (360) was used for gene expression analysis and the expression of each gene normalized to geometric mean of GAPDH and ACTB (relative quantification, RQ). Each bar represents the expression level of specific genes relative to expression of housekeeping genes. An average of four independent experiments and error bars represent the 95% confidence interval (CI) of the mean.

Supplementary Figure 3

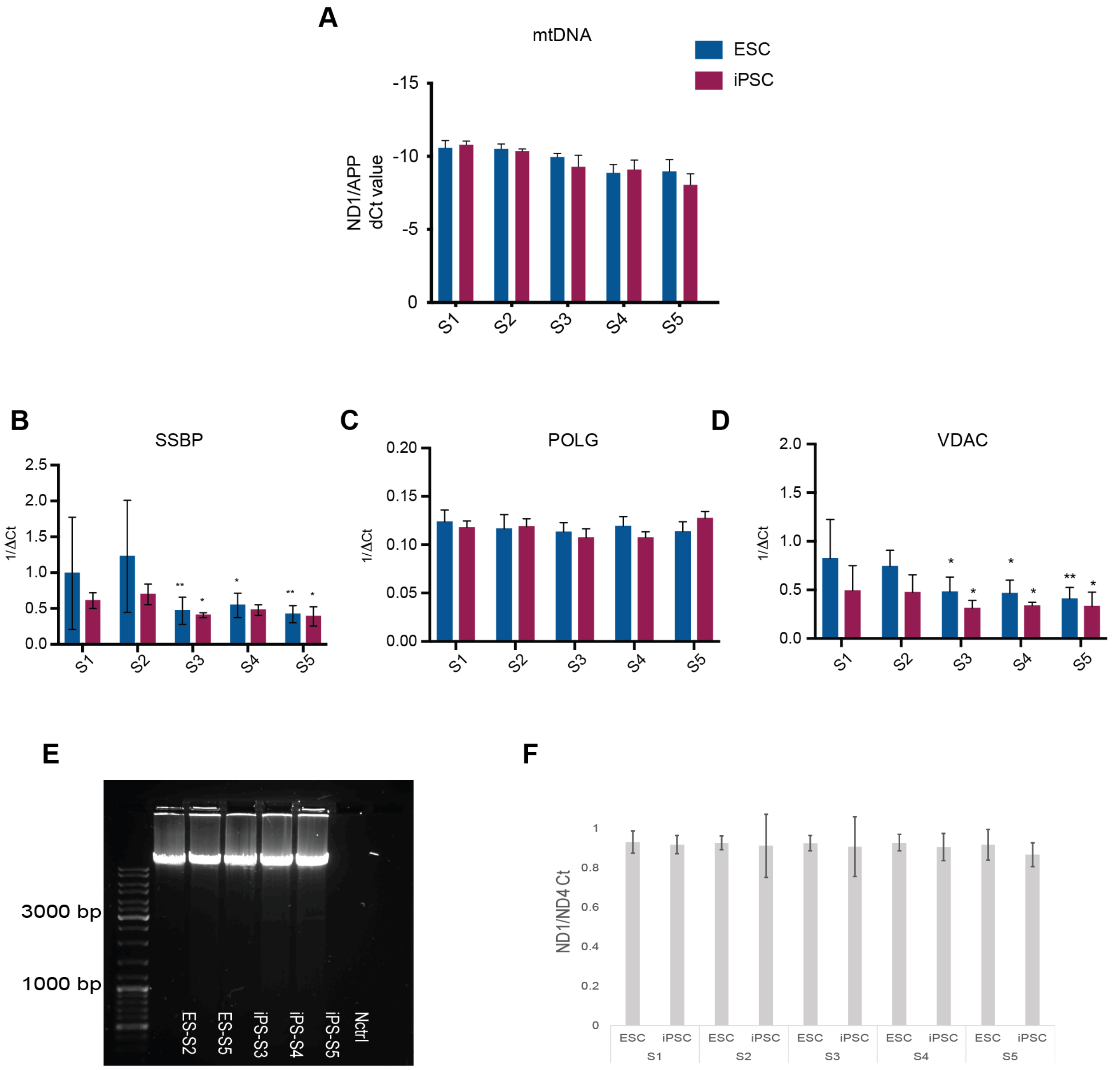

Supplementary Figure 3. Assessing mtDNA replication machinery and integrity during cardiomyocyte differentiation. (**A**) Cycle threshold (Ct) of real-time PCR demonstrated no significant difference in mtDNA level between the two different types of hPSCs (i.e., hiPSC and hESC). (**B, C, and D)** Real-time PCR gene expression analysis for selected markers of the different stages of cardiomyocyte differentiation. HESC (360) was used for gene expression analysis and the expression of each gene normalized to geometric mean of two internal controls (*GAPDH* and *ACTB*). Each bar represents the average of 3–4 independent experiments and error bars represent the 95% confidence interval (CI) of the mean. (**E and F)** We examined the mtDNA integrity of hESCs and hiPSCs in different stages of cardiac differentiation utilizing long-range PCR and real-time PCR, and we could not find any mtDNA deletion.

Supplementary Figure 4

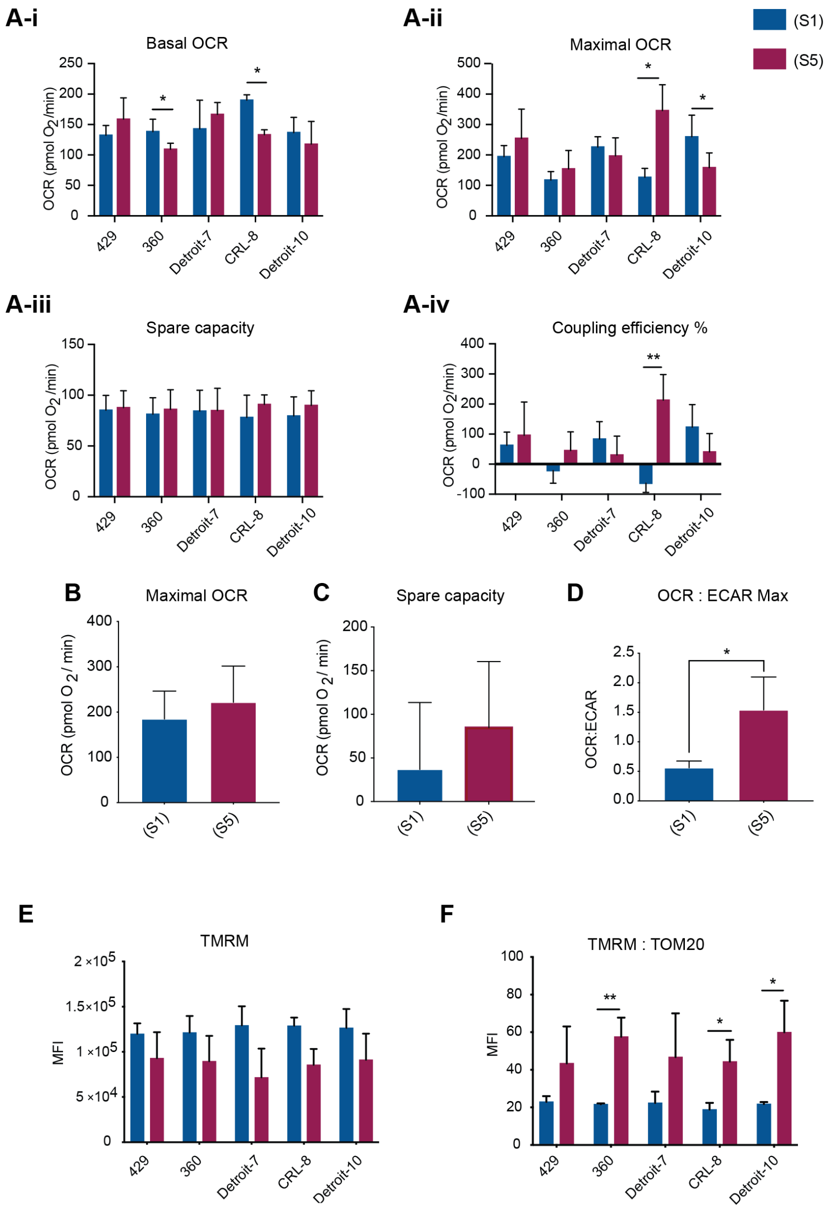

Supplementary Figure 4. Assessing the oxygen consumption rate (OCR) and extracellular acidification rate (ECAR) using a Seahorse XF-96 extracellular flux analyser. (**A)** Bar charts presenting different status of OCR in each individual hPSC line before and after differentiation (S1 vs. S5). (**B and C)** Maximal OCR and spare capacity were showed a slight increase in differentiated cells relative to undifferentiated hPSCs. (**D)** Maximal OCR: ECAR significantly increased, which suggests an increase in mitochondrial respiration activity relative to glycolysis in S5. All of the data collected from the Seahorse experiment normalized to the total protein concentration measured for each well at OD of 280nm. T-tests with Holm-Sidak multiple testing correction was used to assess statistical significance of the difference between S1 and S5 in individual lines. (**E and F)** MFI of TMRM was measured for undifferentiated hPSCs and differentiated cells at S5. We could not detect any significant difference between hESC and hiPSC before or after differentiation. We found significant increase in TMRM level after correction for mitochondrial content. P value= *<0.05, **<0.01, ***<0.001 calculated by Mann Whitney test.

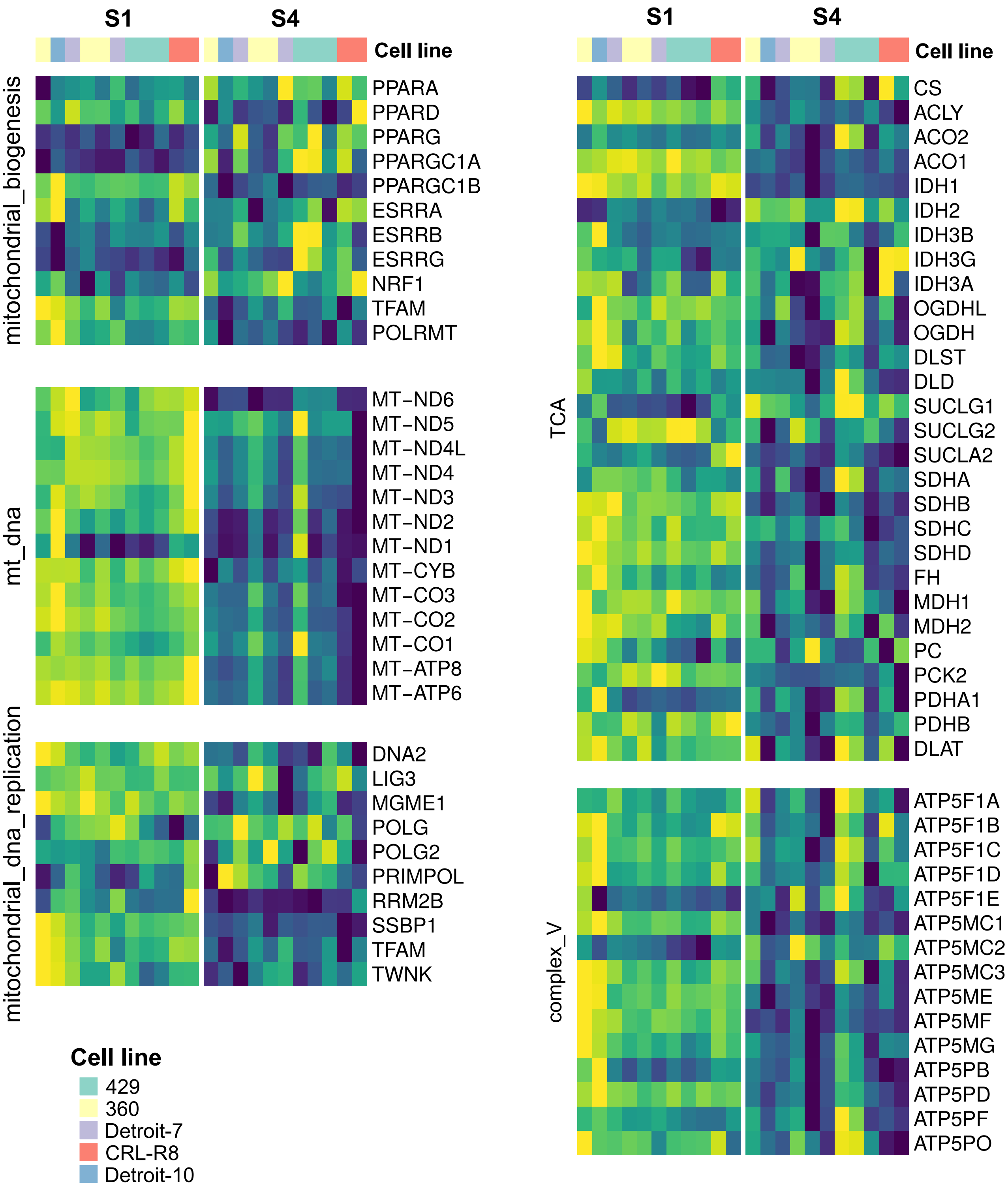

Supplementary Figure 5. Heat map of differentially express genes (S1 versus S4) involved mtDNA homeostasis, mitochondria abiogenesis and ATP-linked activity of mitochondria. 1 is yellow/bright, 0 is blue/dark.

3. Supplementary Movies

Movie 1) Beating cardiomyocytes at S5 generated from hESC (429).

Movie 2) Beating cardiomyocytes at S5 generated from hiPSC (Detroit-7).

Movie 3) Functional cardiomyocytes in the seahorse plate generated from hESC (429).

Movie 4) Functional cardiomyocytes in the seahorse plate generated from hiPSC (Detroit-7).

4. Supplementary Table

Table S1. Reagents

**Flow cytometry antibodies**				
**Marker**	**Host**	**Company**	**Catalog Number**	**Application**
TNNT2	recombinant human	Miltenyi Biotec	130-106-688	Flow Cytometry
SSEA4	Mouse	Thermo Fisher	MA1-021-PE	Flow Cytometry
ISL1	Mouse	bdbiosciences	562547	Flow Cytometry
TOM20	Mouse	Santa Cruz Biotechnology	sc-17764	Flow Cytometry
TFAM	Mouse	abcam	ab198308	Flow Cytometry
**Microscopy antibodies**				
**Marker**	**Host**	**Company**	**Catalog Number**	**Application**
POUF5A	Rabbit	abcam	ab19857	IF
SOX2	Rabbit	abcam	ab97959	IF
NANOG	Rabbit	abcam	ab21624	IF
SSEA4	Mouse	abcam	ab16287	IF
ACTA2	Mouse	abcam	ab7817	IF
CDH5	Rabbit	abcam	ab33168	IF
GJA1	Rabbit	abcam	ab11370	IF
MYL7	Mouse	abcam	ab68086	IF
TNNT2	Mouse	abcam	ab8295	IF
**TaqMan probes**				
**Marker**		**Company**	**Catalog Number**	**Application**
Human GAPD		Thermo Fisher	4333764F	TaqMan Probe
Beta ACT (ACTB)		Thermo Fisher	Hs01060665_g1	TaqMan Probe
POU5F1 (OCT 4)		Thermo Fisher	Hs00999634_gH	TaqMan Probe
T (BRACHYURY)		Thermo Fisher	Hs00610080_m1	TaqMan Probe
MESP1		Thermo Fisher	Hs01001283_g1	TaqMan Probe
ISL1 (ISLET1)		Thermo Fisher	Hs00158126_m1	TaqMan Probe
NKX2-5		Thermo Fisher	Hs00231763_m1	TaqMan Probe
TNNT2 (cTnT)		Thermo Fisher	Hs00943911_m1	TaqMan Probe
VDAC1		Thermo Fisher	Hs01631624_gH	TaqMan Probe
SSBP1		Thermo Fisher	Hs00995376_g1	TaqMan Probe
TFAM		Thermo Fisher	Hs00273372_s1	TaqMan Probe
POLG		Thermo Fisher	Hs00160298_m1	TaqMan Probe

Table S2. Statistical tests description

Experiments	Conditions (stages)	test	N
**MtDNA qPCR**	S1, S2, S3, S4, and S5	Friedman test with Dunn's multiple comparison test	5
**Mitochondria mass**	S1, S4, and S5	Friedman test with Dunn's multiple comparison test	5
**Tfam**	S1, S4, and S6	Friedman test with Dunn's multiple comparison test	5
**Membrane potential**	S1 and S5	Two-tailed unpaired nonparametric t-test (Mann-Whitney test)	5
**Seahorse**	S1 and S5	Two-tailed unpaired t-test with Welch's correction	5

Table S3. RNA-seq Dataset A

Sample ID	Day	Cell line	Type	RIN
1_15S784_S1	D0	CL13L05	hESC	9.8
2_15S872_S6	D0	CL13L05	hESC	10
3_15S952_S11	D0	CL13L05	hESC	8.9
4_15S977_S16	D7	CL13L05	hESC	9.7
5_15S1104_S21	D7	CL13L05	hESC	9.1
6_15S1160_S26	D7	CL13L05	hESC	9.3
7_15S792_S31	D0	CL13L06	hESC	10
8_15S865_S36	D0	CL13L06	hESC	10
9_15S945_S2	D0	CL13L06	hESC	10
10_15S984_S7	D7	CL13L06	hESC	10
11_15S1096_S12	D7	CL13L06	hESC	10
12_15S1152_S17	D7	CL13L06	hESC	9.4
13_15S1416_S22	D0	CL13L25	hiPSC	9.2
14_15S1468_S27	D0	CL13L25	hiPSC	9.8
15_15S1564_S32	D7	CL13L25	hiPSC	8
16_15S1612_S37	D7	CL13L25	hiPSC	8.1
17_15S554_S3	D0	CL14L06	hiPSC	10
18_15S586_S8	D7	CL14L06	hiPSC	9.1
19_15S1425_S13	D0	CL13L20	hiPSC	10
20_15S1477_S18	D0	CL13L20	hiPSC	9.8
21_15S1573_S23	D7	CL13L20	hiPSC	9.2
22_15S1622_S28	D7	CL13L20	hiPSC	7.8

Table S4. RNA-seq Dataset B

Sample ID	Day	RIN
S1_SL403714	D0	9.6
S2_SL403715	D2	8.1
S3_SL403716	D5	7.1
S4_SL403717	D15	8
S5_SL403718	D0	10
S6_SL403719	D2	9.8
S7_SL403720	D5	9.8
S8_SL403721	D15	10
S9_SL403722	D0	9.4
S10_SL403723	D2	7.7
S11_SL403724	D5	8.2
S12_SL403725	D15	7.3

Table S5. List of differentially expressed genes between S1 and S4.

Table S6. List of enriched pathways (GO analyzes) between S1 and S4.

Table S7. List of differentially expressed genes between hiPSC and hESC.

Table S8. List of enriched pathways (GO analyzes) between hiPSC and hESC.
